# Editorial: Environmental factors affecting the germ line epigenome

**DOI:** 10.3389/fendo.2022.1126967

**Published:** 2023-01-05

**Authors:** Alfredo D. Vitullo, Eduardo R. S. Roldan, Candela R. González

**Affiliations:** ^1^ Centro de Estudios Biomédicos Básicos, Aplicados y Desarrollo (CEBBAD), Universidad Maimónides and Consejo Nacional de Investigaciones Científicas y Técnicas (CONICET), Buenos Aires, Argentina; ^2^ Department of Biodiversity and Evolutionary Biology, National Museum of Natural Sciences, Spanish National Research Council (CSIC), Madrid, Spain

**Keywords:** epigenetics, germline, environmental factors, epigenome, gene expression

Environmental influences on the male germ cell epigenome can promote male infertility (1). In recent years, epigenetic mechanisms regulating gene expression have thus raised much interest in reproductive biology, given the possibility of inherited acquired traits through the germ line (2). Environmental factors can induce changes in gene expression without altering DNA sequence through mechanisms including DNA methylation, histone post-translational modifications (PTMs), chromatin remodeling, and non-coding RNAs (2). These mechanisms differentially signal chromatin states promoting open/transcription-permissive or closed/repressed states, or modifying the activity of regulatory elements such as enhancers and promoters (3). Diet, sedentary life, drug consumption or abuse, and exposure to endocrine disruptors may induce stable modifications of the mammalian germline and contribute to trans-generational effects (4). Therefore, much current research is focused on determining how environmental factors influence the germ cell epigenome, if they do, to encode transmissible acquired traits.

This Research Topic gathers several contributions highlighting possible environmental influences on male germ cell epigenome which can result in male infertility.

The first article on this Topic (Faure et al., 2021) shows that *in-utero* exposure to metformin lowers male fertility without changes in sperm production or motility, likely promoted by hypermethylation of genomic DNA associated with a decreased expression of TET1. After birth, we learn through the study of Fenclová et al. (2022), that exposure to endocrine disruptors, such as bisphenols, through maternal milk promotes changes in H3 dimethylation and H2 phosphorylation. These epigenetic changes affect early embryonic development, the quality of germ cells, and thus spermatozoa, which could be the origin of male idiopathic infertility.

González and González put on stage the action of catecholamine as possible mediator between paternal stress responses and alterations of epigenetic marks during spermatogenesis. In their minireview, the authors ask if germ cells can react to catecholamine signaling and encode it in different epigenetic marks in the paternal genome. To tackle this question, they discuss how the testis shares a molecular and transcriptional signature with brain tissue, including a rich expression of catecholaminergic elements in germ cells that seem to respond to stressors with similar epigenetic and transcriptional profiles. Male germ cells express catecholamine receptors at different developmental windows during spermatogenesis, including dopaminergic DRDs and alpha- and beta-adrenergic ADRs receptors. The expression of catecholaminergic components during germ cell maturation may point to these stress hormones as novel epigenetic regulators during spermatogonial and spermiogenic phases. Also, the expression of catecholaminergic components during spermiogenesis, when massive epigenetic events drive chromatin remodeling and nuclear compaction to produce mature spermatozoa, points to post-meiotic germ cells as a vulnerable window for stress-induced epigenetic reprogramming that should be further explored.

In addition to common medical drugs and endocrine disruptors, diet is an environmental factor that can promote a harmful environment for germ cells in mice. A high-energy diet and fat-saturated diets alter spermatogenesis in animal models through gene expression changes lying in epigenome modifications. Soubry et al. explore how healthy versus unhealthy diets are linked to the methylation of imprinted regulatory regions of sperm DNA in humans. They found that a fat-enriched diet increases the chance of sperm methylation at the MEG3-IG CpG4 site. In contrast, diets rich in vegetables and vitamins lowers the risk of DNA methylation at the NNAT CpG3 site. Diet affects sperm epigenetics, opening the possibility of carrying these alterations to the next generation. Should this possibility exist, unhealthy paternal diets could contribute to an increased risk of chronic disease or undesirable metabolic conditions.

Overall, these contributions emphasize that understanding epigenetic regulation in male and female mammalian germ cells will most likely contribute to unraveling coding mechanisms in the transmission of the biological impacts of stress or parental life style between generations.

## Author contributions

All authors listed have made a substantial, direct, and intellectual contribution to the work and approved it for publication.
